# Recycling Attitudes and Behavior among a Clinic-Based Sample of Low-Income Hispanic Women in Southeast Texas

**DOI:** 10.1371/journal.pone.0034469

**Published:** 2012-04-06

**Authors:** Heidi C. Pearson, Lauren N. Dawson, Carmen Radecki Breitkopf

**Affiliations:** 1 Department of Natural Sciences, University of Alaska Southeast, Juneau, Alaska, United States of America; 2 Department of Obstetrics and Gynecology, University of Texas Medical Branch, Galveston, Texas, United States of America; 3 Department of Health Sciences Research, Mayo Clinic, Rochester, Minnesota, United States of America; Public Health Agency of Barcelona, Spain

## Abstract

We examined attitudes and behavior surrounding voluntary recycling in a population of low-income Hispanic women. Participants (*N* = 1,512) 18–55 years of age completed a self-report survey and responded to questions regarding household recycling behavior, recycling knowledge, recycling beliefs, potential barriers to recycling (transportation mode, time), acculturation, demographic characteristics (age, income, employment, marital status, education, number of children, birth country), and social desirability. Forty-six percent of participants (*n* = 810) indicated that they or someone else in their household recycled. In a logistic regression model controlling for social desirability, recycling behavior was related to increased age (*P*<0.05), lower acculturation (*P*<0.01), knowing what to recycle (*P*<0.01), knowing that recycling saves landfill space (*P*<0.05), and disagreeing that recycling takes too much time (*P*<0.001). A Sobel test revealed that acculturation mediated the relationship between recycling knowledge and recycling behavior (*P*<0.05). We offer new information on recycling behavior among Hispanic women and highlight the need for educational outreach and intervention strategies to increase recycling behavior within this understudied population.

## Introduction

Approximately 243 million tons of municipal solid waste, or 4.3 pounds/person/day, were generated in the United States during 2009. However, 82 million tons of waste, or 1.5 pounds/person/day, were recycled in the United States during 2009. This equates to a recycling rate of 34%, a rate which has been steadily increasing since 1965 [1]. Recycling reduces greenhouse gas emissions by lowering the energy (i.e., fossil fuels) required for production of materials and is therefore an important component of efforts to combat climate change [2]. For recycling efforts to be successful, however, widespread public participation is necessary. Thus, it is important to understand factors that influence individual recycling behavior. Knowledge regarding what, how, or why to recycle may lead to increased participation in recycling programs [3]–[9]. Increased access to recycling facilities may also result in increased participation in recycling programs [4], [10]–[13]. In one study, access to recycling services mediated the influence of socioeconomic and demographic variables on recycling behavior [14].

Environmental problems such as waste management have a global distribution but not all nations and cultures address these issues in a similar manner [15]–[17]. Even within a country, recycling behavior may differ according to race/ethnicity [16] and immigrants may be accustomed to waste management and recycling strategies that are different from that found in the U.S. [18]. In particular, an individual's level of acculturation (how closely an individual identifies with his/her country of origin, country of settlement, or both [19], [20]) may affect his/her recycling behavior, as s/he accepts or rejects the cultural ideals and social norms of the U.S.

Although several studies have examined determinants of recycling behavior in predominantly white populations [17], [21]–[23], there is a dearth of information on recycling behavior in Hispanic populations. Few studies have included Hispanics in their sample [13], [16] or have included Hispanics as only a small proportion of the overall sample [17]. As Hispanics represent the largest and fastest growing minority group in the United States [24], it is imperative to understand correlates of recycling behavior within this group. Additionally, recycling behavior in lower socioeconomic or poverty-level samples remains relatively unexplored [13]. The present study fills a gap in the literature by examining correlates of recycling in a low-income Hispanic population.

When examining recycling behavior within a Hispanic population, it is important to consider the effects of birth country and acculturation [17]. Competing views regarding the influence of immigration on environmental concern suggest that foreign-born Hispanics may show either an increase or decrease in recycling behavior compared to U.S.-born Hispanics. One view suggests that individuals born in developing countries will be less likely to engage in pro-environmental behaviors (e.g., recycling) due to immediate concerns for personal economic security that preclude society-level quality-of-life concerns [25]. Another view suggests that environmental degradation in developing countries causes environmental issues to be more salient, leading to an increase in pro-environmental behavior in the immigrant country [25], [26]. The latter view was supported by a study that reported an inverse relationship between acculturation and recycling attitudes, beliefs, and behavior in Hispanics [27]. However, to support or refute either of the aforementioned views, more data are needed to explore the influences of birth country and acculturation on recycling behavior.

When examining recycling behavior within a Hispanic population, it is also important to focus on the sex responsible for household duties and decision-making. In Hispanic families, women are responsible for the majority of household tasks [28] and making decisions related to household matters [29], [30] such as recycling [18]. In general, a targeted study of recycling behavior in women is appropriate as previous studies have reported that women are responsible for carrying out household recycling duties [31] and are more likely to recycle than men [17], [32]. Ultimately, by focusing on women, household recycling behavior may be more accurately described.

The overall aim of the present study was to examine attitudes and behavior surrounding voluntary recycling in a population of low-income Hispanic women. The sample for this study was drawn from southeast Texas, an area where Hispanics comprise approximately 20–50% of the total population [33]. Using a multivariate framework which controlled for social desirability, we examined the relationships between recycling behavior and demographic characteristics, acculturation, knowledge regarding recycling, and potential barriers to recycling (typical mode of transportation, belief that recycling takes too much time).

## Materials and Methods

### Ethics Statement

All procedures performed on subjects were reviewed and approved by the Institutional Review Board of the University of Texas Medical Branch, Galveston under protocol number 05-245 prior to engaging in the research.

### Participants

Participants were 1,512 Hispanic women between 18 and 55 years of age (*M* = 30.32, SD = 8.42). Thirty-seven percent (*n* = 557) were U.S. born, fifty-five percent (*n* = 832) were born in Mexico, seven percent (*n* = 104) were born in Central America, and one percent (*n* = 23) were born elsewhere (Argentina, Peru, Venezuela, Ecuador, Puerto Rico, Dominican Republic, Columbia). One woman did not report her birth country. Fifty percent (*n* = 754) of participants reported an annual household income<$15,000, 48% (*n* = 732) were married, 48% (*n* = 730) reported less than a high school education, and 54% (*n* = 816) did not work outside the home (Table 1). On average, participants had given birth to 2.2±1.36 children (*n* = 1,500, range = 0–9). The median population size of the sample's city of residence was 62,721 (*n* = 1,436). Eight percent (*n* = 127) of participants lived in government-subsidized housing.

**Table 1:** Sampling characteristics by recycling behavior (Yes/No) (*N* = 1,512).

Characteristic	Subcharacteristic	Recycle		Pearson *X* ^2^	df	*P*
		Yes (*n*)	No (*n*)			
Marital status	Unmarried	300	444	20.417	1	<0.001
	Married	381	351			
Income	<$15,000/year	334	420	4.173	1	0.041
	≥$15,000/year	277	277			
Education	<High school	340	390	0.035	1	0.852
	≥High school	354	414			
Employment	Unemployed	401	415	4.893	1	0.027
	Employed	257	338			
Birth country	U.S.	209	348	27.913	1	<0.001
	Outside U.S.	492	462			
Typical transportation	Drives own car	475	555	0.573	1	0.449
	Other	210	225			
Recycling service	None	39	47	15.181	3	0.002
	Curbside	106	105			
	Drop-off	154	255			
	Both curbside and drop-off	355	373			
“I don't know	Disagree	534	542	15.359	1	<0.001
what to recycle”	Agree	168	268			
“Recycling takes	Disagree	599	549	63.369	1	<0.001
too much time”	Agree	103	261			
“Recycling helps save	Disagree	67	113	6.963	1	0.008
landfill space”	Agree	635	697			

### Procedures

Data for the present study were collected between June 1, 2006 and November 9, 2007 as part of an ongoing study addressing cancer prevention. Hispanic, non-Hispanic black and non-Hispanic white women between 18 and 55 years of age who were outpatients in one of four Regional Maternal and Child Health Program (RMCHP) clinics in southeast Texas were screened for eligibility for the main study. Pregnant women, women with a diagnosis of cervical cancer, women who were under the age of 18 or over the age of 55, or who were unable to understand English or Spanish were excluded from participation. If a woman preferred for any reason (including inability to read or write) to have assistance with the survey, the bilingual research assistant that recruited the woman read the survey aloud to the participant and either marked her responses for her, or allowed her to mark her own answers. During the recruitment period captured in the present study, approximately 80% of those meeting eligibility criteria agreed to participate and provided written informed consent for the study. Women were reimbursed $5 for their time.

As part of a comprehensive baseline survey assessing a variety of voluntary behaviors, participants completed a demographic questionnaire and answered several items addressing their knowledge, beliefs and behaviors related to recycling. Participants also completed measures assessing their acculturation and socially desirable response tendencies. Paper and pencil surveys were self-administered and available in English and Spanish. Survey items for which established Spanish translations were unavailable were translated from English to Spanish by a native speaker of Spanish and back-translated by a Spanish–English bilingual individual who possessed knowledge of language nuances typical of Spanish that is spoken in Texas or Mexico. For those items for which an established Spanish translation was available (i.e., for the acculturation measure), the established translation was used. Data from a total of 2,580 women were available on recycling behavior; for this study, secondary data analyses were conducted on the Hispanic subsample which responded to all four recycling questions (*N* = 1,512).

### Measures

#### Recycling knowledge, beliefs, behavior, and service type

Two counter-balanced items assessed knowledge about recycling: “I don't know what to recycle” and “Recycling helps save landfill space.” Response options included a four-point scale ranging from “strongly disagree” (1) to “strongly agree” (4). The first item was reverse-scored relative to the second so that higher scores indicate greater knowledge about recycling. The belief item “Recycling takes too much time” was rated using the same response metric and was reverse-scored so that higher scores indicated a more favorable belief about recycling (i.e., that it did not take too much time). For analysis, each of the four-point scales was collapsed to a 2-point “agree/disagree” scale due to small cell counts in the strongly agree (knowledge items) and strongly disagree (belief item) responses. Cronbach's alpha for the three knowledge and belief items was 0.39 and the mean inter-item correlation was 0.17. Recycling behavior was assessed with the question “Does anyone in your household recycle? (for example, paper, newspaper, magazines, cardboard, glass, aluminum cans, tin cans, plastic)?” Response options for this item were “no” or “yes.” Type of recycling service was determined for each subject's city of residence by information obtained from calling the city hall or reviewing the city's department of public works website. In some cases, direct visual observation of a city's recycling services was conducted due to a lack of information on a city's public works website and/or inability to obtain information from the city hall.

#### Acculturation

A 5-item language-based scale was used to characterize the acculturation level of our Hispanic sample, or the extent to which they ascribe to the dominant, English-speaking culture [34]. Acculturation is related to political and social attitudes as well as a variety of voluntary health and social behaviors. The acculturation scale includes five questions regarding what language the participant uses to read and speak, used as a child, speaks at home, usually thinks in, and speaks with friends. Responses are made using a five-point scale ranging from “only Spanish” (1) to “only English” (5). Using this scale, foreign born Hispanic women should have lower scores than U.S. born Hispanic women. Cronbach's alpha was 0.97 in our sample.

#### Social desirability

Social desirability is similar to impression management and reflects a person's tendency to create a positive image relative to a general tendency to present factual information about the self [35]. Self-reports of a pro-social behavior such as recycling may be vulnerable to the effects of socially desirable responding and few studies account for this effect. The five-item Socially Desirable Response Set (SDRS-5) was used to measure socially desirable response tendency [35]. The scale uses a 5-point “definitely true” to “definitely false” response metric and includes items such as “I sometimes try to get even rather than forgive and forget” and “I am always courteous even to people who are disagreeable.” Social desirability scores are computed such that higher scores reflect a greater tendency toward socially desirable responding. In our sample, Cronbach's alpha was 0.68.

### Statistical Analysis

Descriptive statistics are presented as mean (*M*) ± standard deviation (SD). Independent group t tests were used to determine differences in acculturation according to birth country, and differences in recycling behavior according to age, number of children, and acculturation. Pearson chi-square tests were used to examine associations between recycling behavior and marital status, income, education, employment, birth country, transportation, type of recycling service, recycling knowledge, and recycling belief. Variables that were significant in bivariate analyses were included in the multivariate analysis. Logistic regression was used to determine predictors of recycling behavior while controlling for socially desirable responding. A Sobel test [36] was conducted to determine if acculturation mediated the relationships between the recycling knowledge and belief items and recycling behavior, and if recycling service type mediated the relationships between the recycling knowledge and belief items and recycling behavior. Social desirability and demographic variables that were included in the multivariate analysis were included as covariates in the mediation models. A two-tailed alpha level of 0.05 was considered statistically significant for all analyses. Bivariate and multivariate analyses were conducted using SPSS version 15.0 (Chicago, IL). The Sobel test was conducted using an interactive module available from Preacher and Leonardelli [37].

## Results

Forty-six percent of women (*n* = 702) indicated that they or someone else in their household recycled. Seventy-one percent (*n* = 1076) of women disagreed with the statement, “I don't know what to recycle” and 88% (*n* = 1332) of women agreed with the statement “Recycling helps save landfill space.” Seventy-six percent (*n* = 1148) of women disagreed with the statement “Recycling takes too much time”. Six percent (*n* = 86) of women had no access to recycling facilities, 14% (*n* = 211) had access to curbside services, 27% (*n* = 409) had access to drop-off facilities, and 48% (*n* = 728) had access to both curbside service and drop-off facilities. Typical mode of transportation for 68% of women (*n* = 1,030) was to drive their own car. Foreign-born Hispanic women had lower acculturation scores (*M* = 1.46±0.68), on average, relative to U.S. born Hispanic women (*M* = 4.03±0.90) [*t* (1401) = −60.20, *P*<0.001].

Older women [*t* (1510) = −4.113, *P*<0.001], women with more children [*t* (1498) = −2.900, *P*<0.01], and women who were less acculturated [*t* (1399.754) = 5.972, *P*<0.001] were more likely to recycle. Women who were married (*P*<0.001), had a household income<$15,000/year (*P*<0.05), did not work (*P*<0.05), and were born outside of the U.S. (*P*<0.001) were also more likely to recycle than their respective counterparts (Table 1). Recycling behavior did not vary according to city population size, education, or typical mode of transportation.

Hispanic women living in areas where recycling facilities included both curbside services and drop-off facilities were more likely to recycle than Hispanic women living in areas with no recycling facilities, or in areas with curbside services or drop-off facilities only (*P*<0.01, Table 1). Women who agreed with the statement, “Recycling saves landfill space” were more likely to recycle than women who disagreed with the statement (*P*<0.01). Additionally, women who disagreed with the statements, “I don't know what to recycle” (*P*<0.001) and “Recycling takes too much time” (*P*<0.001) were more likely to recycle than women who agreed with those statements.

The logistic regression model was statistically significant [*X* ^2^ (14, *N* = 1512) = 121.046, *P*<0.001], indicating that the model was able to distinguish between women who did and did not recycle (Table 2). The model explained between 11.2% (Cox and Snell R^2^) and 14.9% (Nagelkerke R^2^) of the variance in recycling behavior, and correctly classified 64.9% of cases. Age, acculturation, and the three recycling items (“Recycling saves landfill space”, “I don't know what to recycle”, “Recycling takes too much time”) were significantly related to recycling behavior while controlling for socially desirable responding. Specifically, Hispanic women who were older and less acculturated were more likely to recycle. Additionally, women who agreed with the statement “Recycling saves landfill space” were 1.7 times more likely to recycle than women who disagreed with this statement. Finally, women who agreed with the statements, “I don't know what to recycle” and “Recycling takes too much time” were 34% and 70% less likely to recycle, respectively, than women who disagreed with those statements.

**Table 2:** Results of logistic regression model for predictors of recycling behavior (*N* = 1,512).

Predictor	B	SE β	*P*	Odds ratio (95% CI)
Constant	−0.576	0.405	0.155	—
Marital status (married)	0.273	0.169	0.106	1.314 (0.943, 1.831)
Income (≥$15,000/year)	0.106	0.146	0.465	1.112 (0.836, 1.479)
Employed	0.098	0.157	0.533	1.103 (0.810, 1.502)
Birth country (U.S.)	0.200	0.248	0.422	1.221 (0.750, 1.986)
Recycling service: None	Ref^a^	Ref	Ref	Ref
Recycling service: Curbside	0.010	0.324	0.976	1.010 (0.535, 1.905)
Recycling service: Drop-off	−0.396	0.300	0.187	0.673 (0.373, 1.212)
Recycling service: Both curbside and drop-off	−0.122	0.293	0.677	0.885 (0.499, 1.571)
“I don't know what to recycle” (Agree)	−0.414	0.159	0.009	0.661 (0.484, 0.902)
“Recycling takes too much time” (Agree)	−1.197	0.175	<0.001	0.302 (0.215, 0.426)
“Recycling helps save landfill space” (Agree)	0.540	0.223	0.016	1.716 (1.107, 2.659)
Age	0.022	0.009	0.019	—
Number of children	0.050	0.059	0.395	—
Acculturation	−0.260	0.089	0.003	—
Social desirability	−0.108	0.043	0.011	—

a Ref = reference group in the analysis.

Acculturation mediated the relationship between the knowledge item, “I don't know what to recycle” and recycling behavior (*z* = 2.19, *P*<0.05). Acculturation did not mediate the relationships between the other recycling items (“Recycling takes too much time”, “Recycling saves landfill space”) and recycling behavior. Similarly, recycling service type did not mediate the relationship between any of the three recycling items and recycling behavior.

## Discussion

We examined correlates of recycling behavior among low-income Hispanic women, an ethnic and socioeconomic group which is understudied with respect to recycling behavior. Similar to previous studies, we found that age [10], [17], knowledge [4], [5], [21], [22], and convenience [10]–[13] exhibited a positive relationship with recycling behavior while acculturation [27] exhibited a negative relationship with recycling behavior. In addition, we failed to find a relationship between income and recycling behavior. One potential explanation is that the low socioeconomic status population from which our sample was drawn provided limited variability in income. Another study based on a sample largely comprising a single socioeconomic class (middle class) also failed to find a relationship between income and recycling behavior [21]. In contrast, Oskamp et al. [22], Gamba and Oskamp [3], and Afroz et al. [38] reported a positive relationship between income and recycling behavior, perhaps due to the increased variability in income present within their study populations.

As suggested by Johnson et al. [17] we included birth country and level of acculturation in our analyses. While birth country was not significant in the multivariate analysis, acculturation exhibited an inverse relationship with recycling behavior and mediated the relationship between recycling knowledge (“I don't know what to recycle”) and recycling behavior. Our results indicate that women who recycled identified more with their native language and culture (Mexico, in most cases) than with U.S. language and culture. In Mexico, women may collect recyclable products for monetary compensation, reuse empty bottles as flower containers, or clothing may be passed between several children within the immediate and extended family [18]. While developed nations typically generate more waste but have more formal, structured recycling programs in place, members of developing nations tend to generate less waste and practice “informal” recycling and reuse initiatives [2]. An additional explanation is that individuals from developing nations with poor environmental conditions are more cognizant of environmental problems and therefore recycle in an effort to protect the environment [26]. As individuals assimilate into U.S. society and become accustomed to the comparatively better environmental conditions in the U.S., the perceived importance of recycling may wane. The results of our mediation model indicated that increased knowledge of recycling held by less acculturated women may have a positive influence on recycling behavior. One strategy for increasing recycling behavior amongst Hispanics may be to encourage retention of traditional pro-environmental values and knowledge about recycling as individuals become more acculturated [27].

This study identified two barriers to recycling: lack of knowledge and inconvenience. Women were less likely to recycle if they did not know what to recycle, did not know that recycling saves landfill space, and believed that recycling takes too much time. These results have important implications for developing educational and intervention strategies to increase recycling behavior within this low-income Hispanic population, where fewer than half of the respondents indicated that they or someone in their house recycled. To increase recycling within this population, one strategy would be to disseminate information explaining what to recycle and the importance of recycling, while also emphasizing the minimal time commitment that household recycling requires. Such information should appear in English and in Spanish, and may be distributed via mailings, signage throughout the community, newspaper ads, or radio/television announcements. Oftentimes, the biggest hurdle in adopting a new behavior is simply overcoming the inertia of getting started [6]. Thus, neighborhood workshops which include guidance and demonstrations of how to clean, sort, store, and transport recyclable material to the appropriate receptacles may also be used to recruit new recyclers, renew commitment among existing recyclers, and generate a “community norm” in which recycling and reusing materials becomes a shared value and normative behavior.

Although the type of recycling service offered (curbside, drop-off, both curbside and drop-off, none) was significantly related to recycling behavior in the bivariate analysis, this relationship was not significant in the multivariate analysis and did not mediate the relationship between the recycling knowledge and belief items and recycling behavior. This suggests that, regardless of the type of recycling service offered, women will recycle if they know what to recycle, the reason for it (it saves landfill space), and do not believe it is a time burden. This reiterates the importance of disseminating informational materials amongst this population, and suggests that allocating resources towards the creation of educational programs (rather than improving the infrastructure of municipal recycling programs) may be an effective strategy for increasing recycling behavior. However, education alone may not be an effective strategy for all populations. For example, a study of low-income minorities in East Harlem, New York found that difficulty accessing recycling services likely exerted a negative effect on recycling behavior [13]. It is possible that the government-subsidized high-rise apartments in which 40% of the East Harlem residents lived created extra logistical barriers to recycling (e.g., inoperable elevators, few recycling containers [13]), whereas most of the women in the present study lived in non-government housing and may not have experienced such logistical barriers to recycling.

While this study provides important information on recycling behavior among Hispanic women, it is not without limitations. First, we used a convenience sample from a larger survey on women's health that was administered in a clinic setting. However, for low-income Hispanic women in south Texas, the clinics from which we recruited are a primary means of healthcare and are known for providing culturally sensitive care to the Hispanic community [39]; thus we believe our sample is an accurate representation of the health-seeking component of the low-income Hispanic population in southeast Texas. Additionally, through our conservative sample selection criteria (i.e., inclusion of only those women who answered all four recycling questions), we attempted to reduce any bias which may have arisen as a result of including the recycling items on an otherwise health-focused survey. Furthermore, although the recycling questions were auxiliary questions to a larger survey on women's health, questions used in the present study occurred on the first three pages of the survey, before the health-specific questions, and were preceded by orienting sentences (e.g., “Now we are going to ask you a few questions about your recycling behavior.”). Second, the measure used to assess recycling behavior was worded so that it included an assessment of recycling behavior of the subject and others in the household. However, as several of the independent variables were relevant to the household as a unit and not the individual subject (e.g., annual household income, typical mode of transportation, marital status) and all analyses concerning the three recycling statements were significant at *P*<0.02, we feel our measures were robust. Additionally, although our sample included only women, there is increasing evidence that Hispanic women are influential decision-makers regarding a variety of household matters [29], [30], [40], [41] and thus a household's behavior is likely to reflect a matriarchal influence. Furthermore, several previous studies have used the term “household” in the measure of recycling behavior [4], [5], [14], [21], [22]. Third, our measure of recycling behavior was based on self-report, which may lead to an upward bias in reported recycling behavior [3]. Including social desirability in the multivariate analysis may have reduced this bias. Finally, inclusion of a low-income, Hispanic, southeast Texas population may limit the generalizability of our findings to populations of different socioeconomic status, ethnicity, and geographic region.

This study fills an important gap in the literature by examining recycling behavior among a population of low-income Hispanic women. However, future studies are warranted that ask more detailed questions regarding recycling behavior within this population. For example, identification of specific gaps in recycling knowledge and an assessment of attitudes, beliefs, and motivations [3], [4] would aid in the development of targeted information for this population. Additionally, while our study design limited the sample to women, it is also important to examine recycling behavior among men, and among a community-based sample. Finally, studies conducted in other geographic regions containing a high percentage of Hispanics (e.g., California, Florida, New York City) will elucidate the generalizability of our findings to Hispanics not of Mexican origin inhabiting other geographic regions.
